# Likelihood-based molecular-replacement solution for a highly pathological crystal with tetartohedral twinning and sevenfold translational noncrystallographic symmetry

**DOI:** 10.1107/S1399004713030319

**Published:** 2014-01-29

**Authors:** Joanna Sliwiak, Mariusz Jaskolski, Zbigniew Dauter, Airlie J. McCoy, Randy J. Read

**Affiliations:** a Center for Biocrystallographic Research, Institute of Bioorganic Chemistry, Polish Academy of Sciences, Noskowskiego 12/14, 61-704 Poznan, Poland; b Department of Crystallography, Faculty of Chemistry, A. Mickiewicz University, Grunwaldzka 6, 60-780 Poznan, Poland; c Synchrotron Radiation Research Section, National Cancer Institute, Argonne National Laboratory, Argonne, IL 60439, USA; d Department of Haematology, University of Cambridge, Wellcome Trust/MRC Building, Hills Road, Cambridge CB2 0XY, England

**Keywords:** maximum likelihood, translational noncrystallographic symmetry, molecular replacement, commensurate modulation, pseudo-symmetry

## Abstract

Translational noncrystallographic symmetry (tNCS) is a pathology of protein crystals in which multiple copies of a molecule or assembly are found in similar orientations. Structure solution is problematic because this breaks the assumptions used in current likelihood-based methods. To cope with such cases, new likelihood approaches have been developed and implemented in *Phaser* to account for the statistical effects of tNCS in molecular replacement. Using these new approaches, it was possible to solve the crystal structure of a protein exhibiting an extreme form of this pathology with seven tetrameric assemblies arrayed along the *c* axis. To resolve space-group ambiguities caused by tetartohedral twinning, the structure was initially solved by placing 56 copies of the monomer in space group *P*1 and using the symmetry of the solution to define the true space group, *C*2. The resulting structure of Hyp-1, a pathogenesis-related class 10 (PR-10) protein from the medicinal herb St John’s wort, reveals the binding modes of the fluorescent probe 8-­anilino-1-naphthalene sulfonate (ANS), providing insight into the function of the protein in binding or storing hydrophobic ligands.

## Abstract 2

With the implementation of a molecular-replacement likelihood target that accounts for translational noncrystallographic symmetry, it became possible to solve the crystal structure of a protein with seven tetrameric assemblies arrayed translationally along the *c* axis. The new algorithm found 56 protein molecules in reduced symmetry (*P*1), which was used to resolve space-group ambiguity caused by severe twinning.

## 1. Introduction   

Hyp-1 is a 165-residue pathogenesis-related class 10 (PR-10) protein from the medicinal herb St John’s wort (*Hypericum perforatum*). PR-10 proteins are among the most mysterious plant proteins since no unique biological function can be attributed to them despite their abundance (Fernandes *et al.*, 2013 ▶). The mystery shrouding the function of PR-10 proteins is in contrast to their comprehensive structural characterization, which reveals an almost hollow molecular core surrounded by a seven-stranded antiparallel β-sheet gripped around a long α-helix (α3) supported at the C-terminus by a fork of two shorter helices (Gajhede *et al.*, 1996 ▶; Biesiadka *et al.*, 2002 ▶). This characteristic fold, termed the PR-10 fold (or the Bet v 1 fold after birch pollen allergen, which was the first PR-10 protein to have its crystal structure solved) strongly suggests the binding/storage of hydrophobic ligands. Such a function would be compatible with signalling and/or regulation, which in plants involve small molecules of diverse structure called phytohormones (Santner & Estelle, 2009 ▶).

Fluorescent probes, such as 8-anilino-1-naphthalene sulfonate (ANS), can be used to study the ligand-binding function of PR-10 proteins in ANS displacement assays (ADAs). To facilitate the interpretation of the spectra, accurate structural information is needed and to this end we have crystallized Hyp-1 in complex with ANS. Hyp-1 has been postulated to catalyze the oxidative coupling of emodin to hypericin, the main pharmacological ingredient of St John’s wort (Bais *et al.*, 2003 ▶), although this enzymatic activity has been questioned (Michalska *et al.*, 2010 ▶). In this context, the binding of ANS, which contains a large π-electron system similar to that of emodin, is of additional interest.

Structure solution by the method of molecular replacement (MR) turned out to be a daunting problem not only because of tetartohedral twinning, but primarily because the asymmetric unit was found to contain multiple copies of the protein molecule arranged with sevenfold noncrystallographic repetition along *c*. This bizarre structural architecture can be interpreted as a superstructure modulation. In crystals with modulated structures, the short-range translational order from one unit cell to the next is lost, but long-range order is restored by a periodic atomic modulation function (AMF; Lovelace *et al.*, 2013 ▶). In general the two periods (of the AMF and of the underlying lattice) can be incommensurate, in which case the superstructure has to be described in a higher-dimensional space (Lovelace *et al.*, 2008 ▶). However, if the modulation is commensurate (as found in this work), it is possible to describe the structure in an expanded unit cell. Superstructure modulation in direct space is manifested in the reciprocal lattice by strong main reflections (from the underlying lattice) and much weaker satellite reflections (from the AMF wave). While superstructure modulation is a well studied phenomenon in small-molecule crystallography, it has been less well studied in macromolecular crystallography. In solving this structure, it was sufficient to consider the structure to arise approximately from a sevenfold replication of the underlying unit cell, and not to be concerned about the details of the changes in orientation and translation described by the AMF. A subsequent publication will address the detailed interpretation of this structure in terms of commensurate modulation.

Note that the word ‘modulation’ is used here in two contexts. In real space, a superstructure modulation causes the atomic positions to vary systematically in different copies in a way that can be represented by a periodic function. In reciprocal space, the repetition of similarly oriented copies causes a modulation of the diffraction intensities, which vary systematically in a way that can also be represented by a (different) periodic function.

## 2. The diffraction data set and initial attempts to solve the structure   

Large single crystals of a Hyp-1–ANS complex were obtained by co-crystallization with an eightfold molar excess of the ligand. Strong blue fluorescence observed under a UV microscope confirmed the presence of ANS in the crystals. X-­ray diffraction data extending to 2.4 Å resolution were collected on the SER-CAT beamline 19ID at the APS synchrotron and were processed with *HKL*-2000 (Otwinowski & Minor, 1997 ▶). The initial merging of the data appeared to be satisfactory in space group *P*422, with an *R*_merge_ of 7.5% (Table 1 ▶). Solvent-content analysis indicated that between six and 12 protein molecules could be accommodated in the asymmetric unit of *P*422.

**Figure 1:**
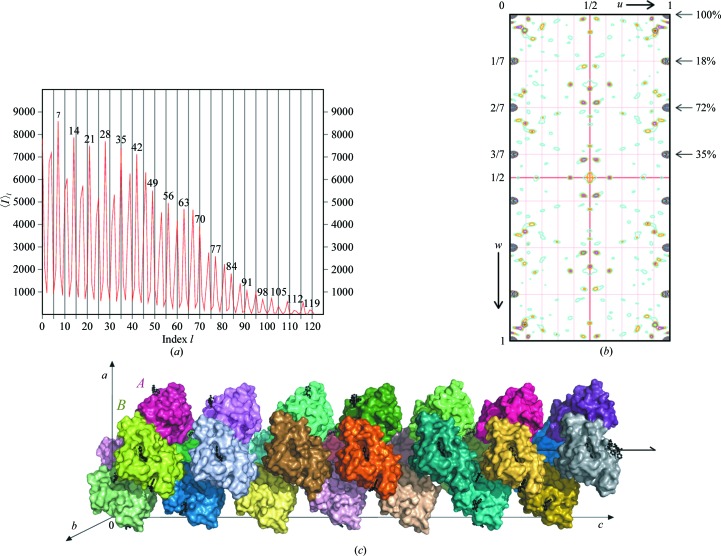
Translational noncrystallographic symmetry in a Hyp-1–ANS crystal. (*a*) Averaged reflection intensities in layers of constant *l* index. The pattern of modulation of the intensities, with peaks separated by 7/2 along c*, is striking. (*b*) Patterson map *v* = 0 section, showing the repetitive peaks (with peak height relative to the origin) along 00*w*. (*c*) The 28 independent Hyp-1 molecules forming the asymmetric unit of the *C*2 crystal packing, arranged in a dimeric pattern with a sevenfold repeat around a noncrystallographic 2_1_ screw (indicated) along the crystallographic *c* direction. Dimer AB is labelled.

The diffraction images revealed a repetitive modulation of reflection intensities along the direction of c* with a period of 7/2 (Fig. 1 ▶ *a*), indicating a noncrystallographic translation of a molecular assembly along the longest cell dimension of the crystal, *c*. In the native Patterson (Fig. 1 ▶ *b*), the peak corresponding to 2/7 of the *c* lattice translation was much stronger (72% of the origin peak height) than the peaks corresponding to 1/7 (18%) or 3/7 (35%) of the *c* axis. In the ultimate crystal structure (Fig. 1 ▶ *c*), these features were shown to arise from an approximate sevenfold repetition of the unit cell along the *c* axis, where molecules separated by 2/7 of the unit cell are generally more similar in orientation than those separated by 1/7 of the unit cell.

Repeated attempts failed to solve the structure by molecular replacement using existing algorithms, even though an excellent model of the unliganded protein was available (Michalska *et al.*, 2010 ▶). We reasoned that the presence of translational noncrystallographic symmetry (tNCS) was violating assumptions in current approaches to molecular replacement, which implicitly assume that the diffraction data vary smoothly over reciprocal space instead of being highly modulated. This structure was therefore used as a test case for new likelihood-based methods taking explicit account of the statistical effects of tNCS.

## 3. Molecular-replacement likelihood function for tNCS   

New likelihood functions that apply corrections for the presence of tNCS were implemented in *Phaser*-2.5.4 (McCoy *et al.*, 2007 ▶). The tNCS is parameterized by the tNCS vector itself and resolution-dependent Luzzati *D* terms (Luzzati, 1952 ▶) that account for deviations in positions between equivalent atoms including the effects of small differences in orientation and small errors in the translation vector. This treatment allows multiple copies of an asymmetric unit substructure to be related by the same tNCS vector, as in this case, in which seven copies are related by approximately the same translation vector. The parameters are used to generate expected intensity factors for each reflection that model the modulations observed in the data (Read *et al.*, 2013 ▶) and are refined against the Wilson distribution (Wilson, 1949 ▶) of the data.

### 3.1. Characterizing tNCS prior to molecular replacement   

The structure-factor contributions from molecules related by tNCS are correlated, with similar amplitudes governed by their similar orientations and with relative phase shifts dependent on the translation vector (Read *et al.*, 2013 ▶). The relative phase shifts create interference effects that modulate the covariances between structure-factor contributions from tNCS-related copies and, consequently, the variance for the total structure factor, thus altering the expected intensities in different parts of reciprocal space. The strength of the modulation is determined by the degree to which the structure-factor contributions are correlated, which in turn is determined by how precisely the conformations and orientations of the tNCS-related molecules or molecular assemblies are preserved. When the multiplicity of the tNCS is high and the orientational differences are effectively random, as for our Hyp-1 crystal, small differences in orientation and relative translation between tNCS-related copies are approximated well by Luzzati *D* parameters (Luzzati, 1952 ▶) describing overall random conformational differences among the molecules, ignoring the small directional dependence of the modulation effects introduced by any rotational differences (Read *et al.*, 2013 ▶). Although we anticipate that the signal in a molecular-replacement search would be stronger if the deviations in the orientations of the tNCS-related copies and in the exact translation vectors relating successive copies could be modelled in advance, we have not yet developed an algorithm that can model such deviations for more than two copies in advance of structure solution.

### 3.2. tNCS correction in molecular replacement   

#### 3.2.1. Covariance elements for true structure factors   

To introduce the notation needed for the application to molecular replacement, we start by briefly reviewing the effect of tNCS on intensity distributions (Read *et al.*, 2013 ▶). For simplicity, in the following we will ignore the effects of measurement errors, but note that these are introduced into the likelihood targets by incrementing the variances in these targets (McCoy *et al.*, 2007 ▶).

The total true structure factor is defined as the sum of contributions from components related by crystallographic (index *k* below) and noncrystallographic (index *m*) symmetry (NCS),
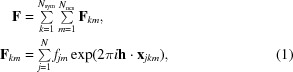
where
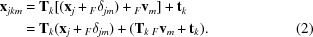



This expresses the idea that all of the tNCS-related copies of a component (with coordinates **x** *_jkm_*) are considered to be derived from a canonical (average) copy centred on the origin (with coordinates **x** *_j_* for unique atom *j*) by a combination of rigid-body translations (translation vector *_F_* **v** *_m_* for NCS copy *m*) with perturbations of both coordinates (perturbation vector *_F_δ_jm_*) and *B* factors (expressed as differences in the scattering factors *f_jm_* for different NCS-related copies). The number of atoms in one copy of the component is given by *N*. In (2), the crystallographic symmetry operator *k* is expressed as a rotation, **T** *_k_*, and a translation, **t** *_k_*. The subscripted prefix *F* indicates a term relating to a component of the true structure factor **F**, to distinguish it from terms relating to the calculated structure factor **G** introduced below.

The expected intensity for a reflection is obtained by adding up all of the covariance elements relating contributions from different components in the unit cell, which are significant for components related by tNCS. The derivation of the expected intensity expression in (3), given in detail in our earlier publication (Read *et al.*, 2013 ▶), is similar to that shown below for the expected values of calculated intensities in (4)–(6) [Disp-formula fd5] [Disp-formula fd6],
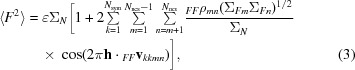
where ∊ is the expected intensity factor arising from crystallographic symmetry, Σ*_N_* is the scattering power of the unit-cell contents, _*FF*_ρ_*mn*_ is the correlation between the tNCS-related structure-factor contributions from components *m* and *n* of the crystal on the same origin, *i.e.* before tNCS translations have been applied (reduced from unity by any perturbations of coordinates or scattering factors), Σ*_Fm_* is the scattering power of one copy of component *m* and _*FF*_ **v**_*kkmn*_ is the translation vector relating the *k*th symmetry copies of components *m* and *n*, analogous to _*GG*_ **v**_*kkmn*_ relating components of the model in (5) below. (3) lacks the *G*-function term (Rossmann & Blow, 1962 ▶) of the expression derived earlier [equation (14) in Read *et al.*, 2013 ▶] because the tNCS-related copies are treated as being in the same orientation. In the notation used here, the subscripted prefix *FF* refers to terms relating the contributions of two components of the true structure factor **F**; below, the subscripted prefix *GG* will be used for terms relating two components of the calculated structure factor **G** and the subscripted prefix *FG* will be used for terms relating one component of **F** to a component of **G**.

#### 3.2.2. Covariance elements for calculated structure factors   

In deriving a likelihood target for tNCS-corrected molecular replacement, the additional covariances relevant to calculated structure factors must also be introduced, including both covariances between tNCS-related contributions to the calculated structure factors and cross-terms between contributions to both the true and calculated structure factors. If it is assumed that the tNCS operations are correctly modelled, then the total calculated structure factors will be governed by modulations similar in size to those of the true structure factors. The same modulations will also apply to terms in the calculation of variances describing the differences between the true and calculated structure factors. Here, we make the approximation that tNCS-related molecules in the model are in an identical orientation and share the same conformation and scattering factors.

As in the case of the true structure factor **F**, the calculated structure factor **G** can be described as the sum over both crystallographic and noncrystallographic symmetry of the copies of contributions from individual models, shown in (4). Note that, without loss of generality, the model and the true structure can be considered to contain the same *N* atoms in each copy of the unique structural motif; atoms present in only one of them can be assigned a scattering factor of zero in the other. The positions of these atoms, denoted **x** in the true structure and **y** in the model, are related by random coordinate errors that will be introduced explicitly later,
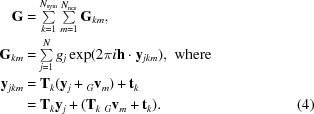



As for (1) and (2) describing the true structure, the coordinates in the model (coordinates **y** *_jkm_* for the copy generated by a combination of symmetry operation *k* and NCS operation *m*) are represented in terms of those from a canonical copy (coordinates **y** *_j_*) of the molecule centred on the origin, translating that copy by a vector *_G_* **v** *_m_* for NCS copy *m*; the major difference from the treatment for the true structure is the lack of the terms describing perturbations of coordinates and scattering factors between the copies. For convenience, we can take the canonical copy to be in the same orientation as the copy with *k* = *m* = 1, so that **y**_*j*_ = **y**_*j*11_ − *_G_* **v**_1_. As for the case of the true structure factor, **F**, we will only consider the covariances between NCS-related molecules in similar orientations which are assumed to be assigned to the same asymmetric unit. The interesting covariances are those between copies related by tNCS (*m* ≠ *n* and *k* = *l*). We can neglect covariances between symmetry-related contributions (*k* ≠ *l*) because these will only be nonzero when the symmetry rotation is parallel to the diffraction vector, and the effect of these will be captured simply by introducing the usual expected intensity factor, ∊.
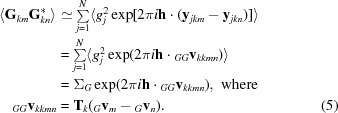



As discussed previously (Read *et al.*, 2013 ▶), terms involving common atoms will dominate, so cross-terms relating different atoms in the NCS copies are ignored in (5). The phase-shift term expressed by the exponential is the same for all atoms, so the sum of squared scattering factors can be factored out as Σ*_G_*, the scattering power of one copy of the tNCS-related component in the asymmetric unit.

The expected calculated intensity is obtained, as for the true intensity, by summing all of the covariance elements,
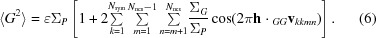



The diagonal elements of the covariance matrix, for which *m* = *n*, are summed in (6) to give Σ*_P_*, the total scattering power of the model. As noted above, the expected intensity factor ∊ accounts for correlations between symmetry-related contributions. Off-diagonal elements of the covariance matrix are paired, and their imaginary components cancel to leave only the cosine term from the phase-shift exponential in (5). The term in the square brackets shows how the overall average intensity, ∊Σ*_P_*, is modulated by the presence of tNCS.

#### 3.2.3. Covariance elements relating contributions to true and calculated structure factors   

The covariance elements relating the contributions to the true and calculated structure factors take the following form:
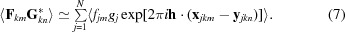



In (7) we assume, as in (5) above, that terms relating common atoms dominate so that there is only a single sum over the unique atoms in a component. We assume that the orientation of the model is correct, on the basis that it will be correct for some orientation in the rotation search, and this orientation should show optimal agreement with the data in the likelihood function. Using the definitions of **F** *_km_* and **G** *_km_* given above, and assuming that the orientations of tNCS-related components in the crystal and the model are identical (with any actual deviations to be modelled by Luzzati *D* factors), the dot product inside the exponential can be expanded, 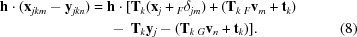



We can simplify this by expressing the coordinates of the model in terms of the true positions of the corresponding atoms in the canonical component of the crystal structure, 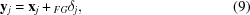
where the random error in the position of atom *j* is given by *_FG_δ_j_*,
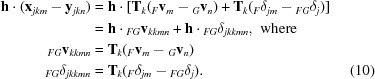



In (10), _*FG*_ **v**_*kkmn*_ is the translation vector relating the *k*th symmetry copies of component *m* in the crystal and component *n* in the model and _*FG*_δ_*jkkmn*_ is the random coordinate error affecting atom *j* in these two components. Substituting (10) into (7) gives (11), 
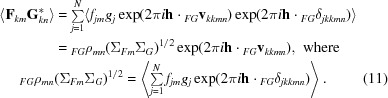



In this equation, the phase-shift term arising from the difference in positions of the component copies, _*FG*_ **v**_*kkmn*_, is the same for all atoms, so it has been factored out. _*FG*_ρ_*mn*_ is the correlation between the structure-factor contributions of component *m* in the crystal and component *n* in the model placed on the same origin (*i.e.* after removing the effect of their relative translation), which is reduced from unity by differences between the coordinates and scattering factors. Note that it can be interpreted as equivalent to a σ_A_ value, as discussed in the context of molecular-replacement ensemble models [equations (14) and (15) of Read, 2001 ▶], so that its value can be estimated in advance of structure solution from the expected r.m.s. error of the model (estimated in turn from the sequence identity and size of the model; Oeffner *et al.*, 2013 ▶) and the completeness of the model.

#### 3.2.4. Conditional probability distribution given a model   

The conditional probability of the true structure factor given a model is obtained most easily by starting from the joint distribution of all of the NCS-related contributions to the true and calculated structure factors. This is similar to the strategy used to derive likelihood functions for molecular replacement (Read, 2001 ▶) and experimental phasing (Read, 2003 ▶). A large covariance matrix, Σ, is partitioned into separate matrices for the contributions to the true structure factor (Σ_11_), the contributions to the calculated structure factor (Σ_22_) and the covariances between them (Σ_12_ and Σ_21_, related by a Hermitian transpose). The individual submatrices have a block-diagonal structure, with blocks reflecting the correlations among copies related by translational NCS and zeroes for the symmetry-related copies that (after accounting for the crystallographic expected intensity factor ∊) can be considered uncorrelated.
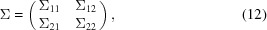


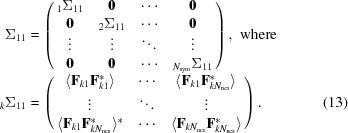


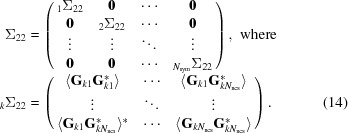


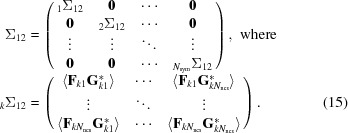



Because the covariance matrix has Hermitian symmetry, Σ_21_ = Σ*^H^*_12_. 

The matrix manipulations used to derive the conditional distribution require inverting the Σ_22_ submatrix and then computing products with the off-diagonal submatrices. Note that the inverse of a block-diagonal matrix is itself a block-diagonal matrix, in which the individual blocks (denoted by a subscripted prefix) are the matrix inverses of the original blocks.
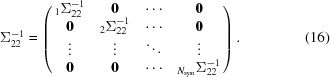



In addition, the product of two block-diagonal matrices is itself a block-diagonal matrix, in which the individual blocks are the products of the corresponding blocks from the original matrices, 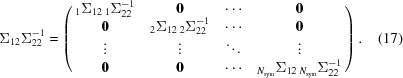



Thus, all of the manipulations used to derive the conditional probability distributions involve operations carried out only on the blocks corresponding to the NCS-related contributions to a particular symmetry copy in the crystal and the model.

#### 3.2.5. Conditional probability when the rotational component of the tNCS operator is zero   

The terms in the submatrix block _*k*_Σ_12_, *i.e.* 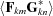, can be related to the terms in the submatrix block _*k*_Σ_22_, *i.e.* 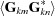, if we make some reasonable assumptions. The guiding principle is that if we had a clear idea of the systematic differences between the model and the true structure then we would have changed the model accordingly, so any differences that remain should be random. If the NCS translations in the true structure and the model were identical, then the exponential phase-shift terms in (5) and (11) would be identical, giving
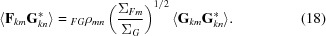



Considering the interpretation of _*FG*_ρ_*mn*_ as a σ_A_ value, as discussed in §[Sec sec3.2.3]3.2.3, and noting the definition of σ_A_ in terms of model completeness and the Luzzati (1952 ▶) *D* factor (Srinivasan & Ramachandran, 1965 ▶), where
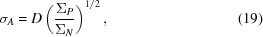
(in which Σ*_P_* plays the same role as Σ*_G_*, and Σ*_N_* plays the same role as Σ*_Fm_*), we obtain a simple relationship between the terms in the submatrix block,
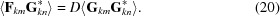



If we assume that the tNCS translations in the true structure and the model differ instead by a random error that is independent of the model errors, then the correlation between the true and calculated structure-factor contributions will be somewhat lower, which can be modelled by assuming a slightly larger r.m.s. error in computing the values of *D* as a function of resolution. Note that the effective r.m.s. errors are refined as part of the final step of molecular replacement in *Phaser*.

The same errors should apply to different components, so we can approximate the whole off-diagonal submatrix blocks as
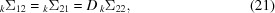
so that
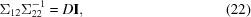
where **I** is an identity matrix.

With these results in hand, standard manipulations can be applied to obtain the expected values of the symmetry- and NCS-related contributions to the true structure factor, given the corresponding contributions from the model,
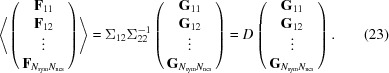



In words, the expected values of the various contributions **F** *_km_* to the total structure factor are simply the calculated contributions **G** *_km_* multiplied by *D*. The covariance matrix expressing the uncertainties in those expected values is 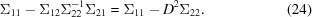



For the probability distribution of the total true structure factor, the variance is given by the sum of the elements of this updated covariance matrix, and the expected value is simply *D* times the total calculated structure factor. For acentric and centric reflections, the structure-factor probability distributions are thus given by
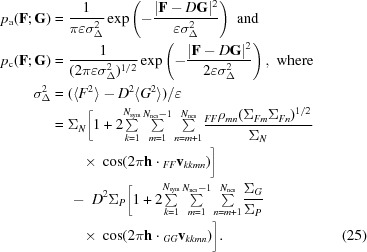



In the general expression for σ_Δ_^2^, it would be possible for one of the terms to be more highly modulated than the other. If care were not taken with the parameterization or with constraining the relative values of different terms (especially *D*), then this variance term could become negative. In practice, the modulation factors applied to the true and calculated intensities can often be assumed to be equivalent.

We will consider elsewhere the effects of modelling the rotational differences when there are only two tNCS-related copies and the approximations inherent in the treatment presented here are poorly satisfied.

## 4. Hyp-1 tNCS-corrected molecular replacement   

### 4.1. Attempts in *P*422-type symmetry   

Molecular-replacement searches were carried out in *Phaser*-2.5.4, which included the likelihood functions able to account for the intensity modulations owing to translational NCS described above. Refinement of the tNCS operators relating pairs of molecules in space group *P*422 gave an optimal translation vector of (−0.004, −0.004, 0.285). (Note that the statistical effects of the tNCS operators depend only on the point group, but not on the particular space group.) Searches were carried out in all primitive space groups with 422 point-group symmetry, looking for seven copies related by tNCS. Using Hyp-1 as a model (Michalska *et al.*, 2010 ▶), multiple non-equivalent solutions with high signal to noise were found for space group *P*4_1_22, showing similar but non-identical packing. However, space group *P*4_1_22 is ruled out by the presence of strong 00*l* reflections where the index *l* is not a multiple of 4. This fact, the existence of multiple incompatible solutions and the failure of the model to refine to an *R* factor better than 48% all suggested that the crystal was pseudo-symmetric, with the true symmetry being lower than *P*422. However, the excellent merging statistics in *P*422 suggest that if the crystal is pseudo-symmetric it is also twinned. In agreement with this, the *L* test (Padilla & Yeates, 2003 ▶) suggested the presence of twinning; when reflections offset by multiples of 2 in *h* and *k* and multiples of 7 in *l* were used for the *L* test, the values 〈*L*〉 = 0.458 and 〈*L* ^2^〉 = 0.288 were obtained. Pseudo-symmetry and twinning are commonly found in conjunction with one another (Lebedev *et al.*, 2006 ▶), and the presence of pseudo-symmetry would explain why the intensity distributions are perturbed less than one would otherwise expect for perfect twinning, where 〈*L*〉 = 3/8 and 〈*L* ^2^〉 = 1/5, compared with 〈*L*〉 = 1/2 and 〈*L* ^2^〉 = 1/3 for untwinned data.

### 4.2. Structure solution in space group *P*1   

To identify the true symmetry, the diffraction data were expanded to *P*1 and molecular replacement was attempted looking for 56 copies of Hyp-1. It can be difficult to resolve cases of pseudo-symmetry because if a perfectly symmetric solution is generated the symmetry has to be broken in some way, but the symmetric solution is balanced between different ways in which the symmetry can be broken. To avoid this trap, the search in *P*1 was carried out in a way designed to avoid perfect symmetry, particularly the sevenfold translational pseudo-symmetry. A search for the first molecules in *P*1 was carried out by assuming that the second through seventh molecules would be generated from the first by successive applications of the translation vector (−0.004, −0.004, 0.285), as revealed by refinement of the tNCS operators in the 422 point-group symmetry (see above). After rigid-body refinement of the top solution, seven additional copies of this assembly of seven molecules were added to yield a solution with 56 copies of Hyp-1 in the unit cell.

### 4.3. True space group identified as *C*2   

Rigid-body refinement of the solution with 56 copies of the protein molecule in the *P*1 unit cell was carried out using *phenix.refine* (Afonine *et al.*, 2012 ▶). To determine whether the molecular-replacement solution obeyed higher symmetry than *P*1, the calculated structure factors were examined for evidence of symmetry using *POINTLESS* (Evans, 2006 ▶), which looks for agreement between structure factors related by potential symmetry operators of the lattice. Only one of the diagonal dyads of the initial *P*422 space group ([110] direction of the tetragonal lattice) gave good agreement between related structure factors. This twofold operator corresponds to the unique *y* direction of space group *C*2, following the reindexing operation (*h* + *k*, *k* − *h*, *l*).

Accordingly, the diffraction data were reprocessed in the correct *C*2 symmetry, with the results presented in Table 1 ▶. Unfortunately, the data-collection strategy had been selected for tetragonal symmetry, and instead of covering the unique 90° of rotation (between directions parallel and perpendicular to the monoclinic twofold axis) necessary for completeness, the same (*i.e.* symmetry-equivalent) 45° region of reciprocal space was covered twice. This led to a completeness of only ∼73% in the genuine monoclinic symmetry. Since the *R*_merge_ value for *P*422 (7.5%) was only less than 1% higher than that for *C*2 (6.6%), with much higher multiplicity, it was decided to exploit this effect of the crystal twinning and to use in all subsequent calculations a data set expanded from *P*422 to *C*2 symmetry. This data set is almost fully complete and has the same statistical characteristics as presented in the first column of Table 1 ▶. Since the intensities conform to 422 symmetry, they correspond to a pseudo-tetartohedrally twinned crystal. The twinning of the monoclinic data set thus obtained is perfect, although in the real crystal it might have been only nearly perfect.

### 4.4. Structure solution in space group *C*2   

The *C*2 data were used to solve the structure by molecular replacement again, searching for four copies of the set of seven protein molecules found in the first step of the *P*1 structure solution. This yielded two clear solutions with identical likelihood scores. Although the two solutions were not crystallographically equivalent, they were related by a fourfold rotation corresponding to one of the tetartohedral twin operators for *C*2. Rigid-body refinement of the 28 copies of the protein molecule in the *C*2 solution confirmed that this solution does not obey any higher symmetry, though it is pseudo-symmetric with pseudo-tetragonal symmetry. The fact that the data could be merged well in point group 422 indicates that the additional apparent symmetry arose from twinning (Lebedev *et al.*, 2006 ▶).

## 5. Refinement of the structure   

**Figure 2:**
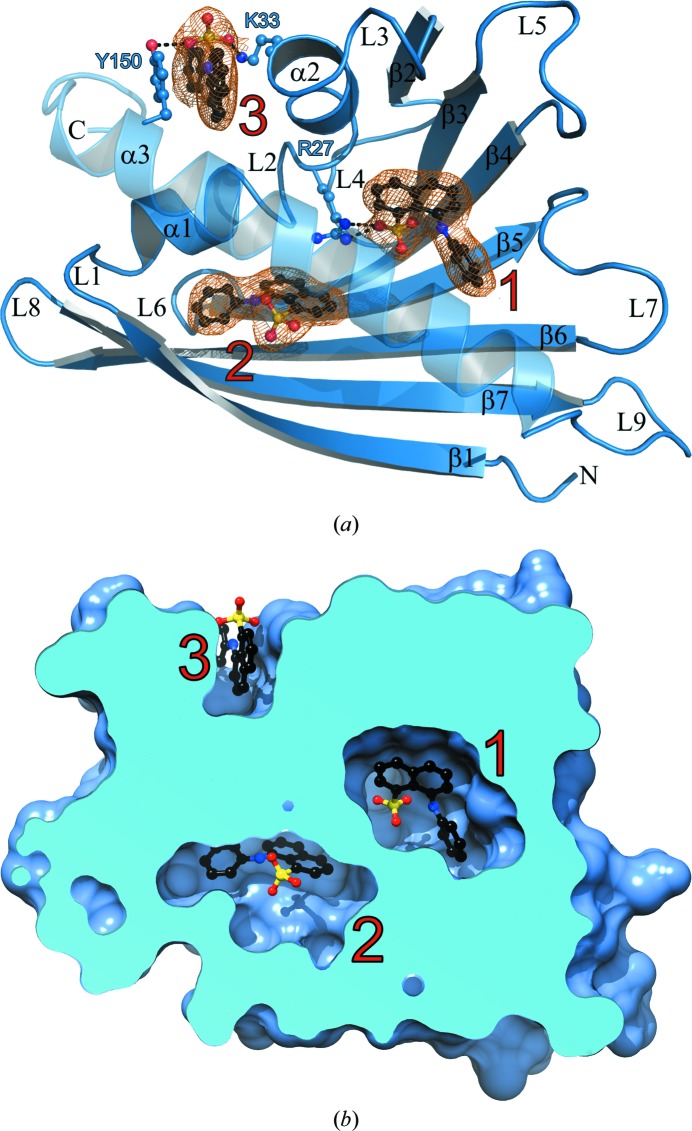
ANS binding to copy *K* of Hyp-1. (*a*) 2*F*_o_ − *F*_c_ electron density contoured at 1.5σ around the ligands, showing the ANS molecules (red labels). Two ligands are bound in internal chambers (sites 1 and 2) and one in a deep surface pocket (site 3) formed by residues Lys33 and Tyr150. Sites 1, 2 and 3 are occupied in 22, 25 and 13, respectively, of the 28 protein molecules in the asymmetric unit. Dashed lines indicate hydrogen bonds to protein atoms. The ribbon diagram is annotated with numbered secondary-structure elements, with α for helices, β for β-strands and L for loops. (*b*) A cutaway view of protein molecule *K* generated with *Chimera* (Pettersen *et al.*, 2004 ▶), showing ligand positions relative to the protein surface.

Before the atomic coordinate refinement commenced, data were selected for *R*_free_ tests using *SHELXPRO* (Sheldrick, 2008 ▶) within narrow shells of resolution in order to guarantee the inclusion of NCS-related reflections. The structure was refined in *REFMAC*5 (Murshudov *et al.*, 2011 ▶) with intensity-based twin detection/refinement and jelly-body refinement. As expected from the molecular replacement and the treatment of the intensity data, four twin domains were found with operators corresponding to the twofold axes of the tetragonal supersymmetry. Upon refinement, all of the twin fractions converged at about 0.25. Application of loose NCS restraints to all 28 independent copies of the Hyp-1 molecule resulted in a slight improvement of the refinement statistics. In the final refinement, the NCS restraints were removed without any effect on the refinement statistics. *REFMAC* refinement was alternated with manual rebuilding in *Coot* (Emsley *et al.*, 2010 ▶). After modelling 89 ANS molecules and 35 water molecules, the final refinement converged with *R* and *R*_free_ factors of 22.2 and 27.7%, respectively. The r.m.s. deviation from standard bonds was 0.015 Å, with 91.8% of all residues in favoured and 7.0% in allowed Ramachandran regions and just a few Ramachandran outliers in loops L4 and L7, which were partially disordered. The final electron-density maps are of very good quality, showing unambiguously the main-chain trace of all 28 independent protein molecules (*A*, *B*, … *Z*, *a*, *b*), clear conformations for most side chains and good density for all copies of the C-terminal helix α3, which is often disordered in PR-10 structures. In addition, the 89 ANS molecules have very good definition in the electron density (Fig. 2 ▶ *a*).

## 6. Ligand binding by Hyp-1   

The maps show excellent electron density for either one, two or three internal ANS molecules (at sites designated 1, 2 and 3) per Hyp-1 protein (Fig. 2 ▶) and 29 interstitial ANS molecules. This structure of the Hyp-1–ANS complex therefore has implications for the ADA method of studying ligand binding to PR-10 proteins using fluorescent probes. The structure shows three clearly defined and separated ligand-binding sites, and the fact that the complex stoichiometry can be 1:1, 1:2 or 1:3 has to be taken into account as a complication when studying the kinetics and stoichiometry of PR-10–ligand complexes using ANS displacement fluorescence. Fortunately, the structure shows that there is no direct interaction between the fluorescing species to further complicate the spectra.

## 7. Crystal packing and superstructure modulation   

The Hyp-1 molecules are arranged into dimers through intermolecular β-sheet formation between β1–β1 strands, although the protein is monomeric in solution. Seven of these dimers have the same orientation and nearly equal repetitive spacing along the *c* axis, while the remaining seven are their copies through a noncrystallographic 2_1_ axis in the *c* direction. This packing arrangement creates a noncrystallographic screw axis with ∼180° rotation and 1/14 translation (Fig. 1 ▶ *c*). The interstitial ANS molecules have a similar but not identical disposition with respect to the sevenfold symmetric packing of the protein molecules. This variation explains why the crystal has a unit cell with a pseudo-sevenfold translation along the *c* axis instead of a smaller cell.

The peculiar pattern of reflection intensities in the c* direction and the repetitive pattern of molecular packing in the corresponding direction in direct space, leading to a sevenfold expansion of the basic unit cell, are both strong indications that we have a case of a modulated superstructure. Since it was possible to successfully refine the structure using a sevenfold expanded unit cell, the modulation appears to be commensurate. Modulated structures have been well studied in small-molecule crystallography but are practically unheard of in macromolecular crystallography (Porta *et al.*, 2011 ▶). These aspects of the Hyp-1–ANS crystal structure will be treated elsewhere.

## 8. Conclusion   

Our crystal form of the Hyp-1–ANS complex is a case of a modulated superstructure. In protein crystallography such reports are rare (Porta *et al.*, 2011 ▶), most likely not because such cases do not exist but because such crystal structures are rejected as too difficult to solve. The present modulation is evidently commensurate, which allows its description in a larger unit cell (here, repeated sevenfold along *c*) without having to resort to description in a higher-dimensional space (Wagner & Schönleber, 2009 ▶), which would be very difficult indeed.

In this study, we have demonstrated that novel maximum-likelihood algorithms that account for the structure-factor modulations induced by tNCS are extremely powerful in tackling even the most difficult cases in macromolecular crystallography. In this particular example, the algorithm correctly located 56 copies in space group *P*1 of the protein molecule used as a probe, despite near-perfect tetartohedral twinning. The success of our approach is important as it shows that modulated macromolecular superstructures do not have to be discarded but can in fact become sources of structural information on a par with unmodulated structures. Finally, the particular ANS complex of a PR-10 protein shows at atomic detail unexpected protein interactions that have to be taken into account when using ANS as a fluorescent probe in studies of biologically relevant ligand molecules.

The version of *Phaser* that accounts for tNCS using the algorithms described here is available as part of the current releases of both the *CCP*4 (Winn *et al.*, 2011 ▶) and *PHENIX* (Adams *et al.*, 2010 ▶) packages.

## Figures and Tables

**Table 1:** Diffraction data statistics Values in parentheses are for the highest resolution shell.

Beamline	19ID, SER-CAT, APS	
Temperature (K)	100	
Space group	*P*422	*C*2
Unit-cell parameters		
*a* (Å)	103.42	146.21
*b* (Å)	103.42	146.12
*c* (Å)	298.50	298.35
β (°)	90	90.07
Wavelength (Å)	1.000	1.000
Resolution (Å)	30–2.43 (2.47–2.43)	30–2.43 (2.47–2.43)
Reflections, measured	496579	495931
Reflections, unique	61810	170447
Completeness (%)	99.8 (99.2)	72.7 (65.9)
〈*I*/σ(*I*)〉	26.4 (2.6)	13.4 (1.5)
*R*_merge_ † (%)	7.5 (75.8)	6.6 (69.1)
Multiplicity	8.0 (7.1)	2.9 (2.6)

† *R*_merge_ = 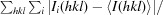 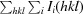.
